# Changes in Membrane Receptors and Ion Channels as Potential Biomarkers for Osteoarthritis

**DOI:** 10.3389/fphys.2015.00357

**Published:** 2015-12-01

**Authors:** Rebecca Lewis, Richard Barrett-Jolley

**Affiliations:** ^1^Faculty of Health and Medical Sciences, School of Veterinary Medicine and Science, University of SurreyGuildford, UK; ^2^Department of Musculoskeletal Biology, Faculty of Health and Life Sciences, Institute of Ageing and Chronic Disease, University of LiverpoolLiverpool, UK

**Keywords:** ion channels, osteoarthritis, biomarkers, chondrocytes, cartilage

## Abstract

Osteoarthritis (OA), a degenerative joint condition, is currently difficult to detect early enough for any of the current treatment options to be completely successful. Early diagnosis of this disease could increase the numbers of patients who are able to slow its progression. There are now several diseases where membrane protein biomarkers are used for early diagnosis. The numbers of proteins in the membrane is vast and so it is a rich source of potential biomarkers for OA but we need more knowledge of these before they can be considered practical biomarkers. How are they best measured and are they selective to OA or even certain types of OA? The first step in this process is to identify membrane proteins that change in OA. Here, we summarize several ion channels and receptors that change in OA models and/or OA patients, and may thus be considered candidates as novel membrane biomarkers of OA.

## Introduction

Early diagnosis of osteoarthritis (OA) is difficult as significant joint damage generally occurs before patients present with pain. MRI screening of risk groups for markers of joint degeneration, by definition, requires that some degeneration has already taken place. Although, there is a clear correlation between the presence of cartilage-denuded zones of subchondral bone in knee joints and pain severity (Cotofana et al., 2013), the overall correlation between physical indicators of joint degeneration and osteoarthritic pain is statistically significant, but weak (Hunter et al., 2013; Eckstein et al., 2015) therefore alternative markers of early OA are urgently needed. Membrane proteins are a potential source of novel biomarkers. A normal human cellular membrane contains thousands of proteins, so this review will focus largely on ion channels and membrane proteins involved with intercellular signaling. Here we mainly consider the cartilage-producing cells; chondrocytes. There are many other cell types that are also likely to be important for development of OA, such as those of bone, blood vessels, synovial tissue or nerves, but these are beyond the scope of this review.

## Membrane proteins as biomarkers of osteoarthritis

The term “biomarker” is often misappropriated to mean *just* soluble biomarkers useable for diagnostics, but in fact the WHO organization defines the term much more broadly “almost any measurement reflecting an interaction between a biological system and a potential hazard, which may be chemical, physical, or biological. The measured response may be functional and physiological, biochemical at the cellular level, or a molecular interaction.” (Strimbu and Tavel, 2010). Plasma membrane proteins could therefore constitute useful biomarkers in a number of contexts. Firstly, recent developments of adaptamer and nanotechnologies (Gao et al., 2004; Hwang et al., 2010) have demonstrated that changes in cellular membrane protein components or even those of intracellular compartments can be detected *in vivo*. Whilst this approach would be more challenging in the hypocellular and avascular environment of cartilage, it may prove possible especially in synovial tissue. As imaging technology becomes more widely available, we need to have potential biomarkers available for it to exploit. Secondly, there are a number of conditions in which membrane proteins, or parts of membrane proteins, are shed and become detectable as soluble biomarkers. For example, in liver cirrhosis, the aquaporin channel (AQP2) is increased both in expression and in urinary excretion (Asahina et al., 1995; Ivarsen et al., 2003; Pedersen et al., 2003). During progression of OA, fragments of the membrane protein syndecans can be detected in synovial fluid (Pap and Bertrand, 2013). A third context in which an expanded knowledge of membrane receptors will prove useful is in genetic screening. It is unlikely that any ion channel or receptor will be found that is unique to components of the joints; if a change in a membrane receptor or channel is detected which is involved with OA and arises from, for example, a genetic polymorphism, it is logical that this could then be detected with a blood test. Indeed the Na_*V*_1.7 ion channel has already been identified as a potential biomarker of OA in genetic association studies (Thakur et al., 2013). Whilst OA is a complex multi-organ condition, many studies use so called *in vitro* models of OA however these are largely unvalidated (Johnson et al., in press); a further, potentially valuable use of membrane biomarkers will be to more precisely characterize these models and compare their differential membrane phenotype with that of tissue from native OA cartilage.

## Differentially expressed channels and receptors in osteoarthritic cartilage

The vast majority of studies investigating changes in membrane receptors and ion channels in OA have focussed on chondrocytes, the resident cells of cartilage that detect activity of the joints and respond with production and maintenance of further cartilage (Urban, 1994). Sudden impact loading of joints can damage chondrocytes and will decrease cartilage production (Quinn et al., 2001; Milentijevic et al., 2003; Bush et al., 2005; Natoli et al., 2008), but paradoxically, joint inactivity also leads to reductions in cartilage production (Brandt, 2003). Thus, there appears to be an optimal chondrocyte-loading regime. The frequency of loading and amount of loading are unknown. Evidence suggests that this is disturbed in OA (Millward-Sadler et al., 2000; Vincent, 2013) and so elements of the mechanotransduction system are potentially key sources of novel membrane biomarkers. Chondrocyte mechanotransduction is poorly understood, but the membrane proteins; integrins, connexins, TRP, piezo, ENaC, and potassium channels have been strongly implicated (Millward-Sadler et al., 2000; Mobasheri et al., 2002; Garcia and Knight, 2010; Guilak, 2011; Lewis et al., 2011b, 2013a; O'Conor et al., 2013; Lee et al., 2014) in addition to the soluble mediator, FGF2 (Vincent et al., 2007).

### Ion channels

In a recent report, we discussed the differential expression of ion channels in OA (Lewis et al., 2013b). We analyzed transcript levels in the (Karlsson et al., 2010) dataset; the acid sensing potassium channel (TASK-2), epithelial sodium channel (ENaC) and Ca^2+^ activated chloride channel were all decreased (anoctamin-1, TMEM16), whereas Ca^2+^ activated potassium channels (KCa3.1, “SK” and KCa1.1, “BK”) and aquaporin 1 (AQP1) were strongly up-regulated. The tight clustering of differentially expressed channels to ontological functions of mechanotransduction, cell volume regulation and apoptosis suggests that these changes could be linked to progression of OA. To further investigate this channel data we analyzed protein expression of BK in osteoarthritic cartilage by immunohistochemistry and aquaporin expression using a functional (permeability) assay. Both aquaporin and BK were significantly increased in expression in chondrocytes from osteoarthritic cartilage (Lewis et al., 2013a,b). Increased aquaporin channel expression in OA has also been reported elsewhere (Geyer et al., 2009; Hagiwara et al., 2013; Musumeci et al., 2013) and the AQP1 gene harbors hypomethylated regions of DNA in OA patients indicative of over-expression (Rushton et al., 2014). This striking observation raises the possibility of there being changes in other detectable partners in the volume-regulatory pathway, such as water content, potassium or any of several other cellular markers (Hoffmann et al., 2009). Changes in synovial fluid osmolarity during progression of osteoarthritis could also influence progression of the disease due to the effects on ion channel expression. The ClC7 chloride channel, for example, is downregulated by hypo-osmotic stress, altering membrane potential and leading to increased cell death (Kurita et al., 2015). Another potassium channel, not identified as differentially expressed in our transcriptomic analysis, but linked to OA by more traditional methods, is the ATP sensitive K^+^ ion channel (K_ATP_). K_ATP_ is a widely expressed ion channel, existing in several isoforms and involved in many human diseases. In our own work we identified K_ATP_ channels in chondrocytes (Mobasheri et al., 2007) and a further recent report showed this channel is linked to control of chondrocyte metabolism in a scheme involving the glucose transporter family GLUT-1 and GLUT-3 (Rufino et al., 2013). This function of K_ATP_ channels is changed in OA and implicates changes in chondrocyte metabolism in the complex process of cartilage degeneration.

TRP cation channels are a widely distributed family of channels in the musculoskeletal system that frequently detect changes in the cellular microenvironment and transduce these to electrochemical signals (Guilak et al., 2010). In our own studies we reported that TRPV5 channels were present in healthy chondrocytes and facilitated the volume defense mechanism of chondrocytes (Lewis et al., 2011a; Hdud et al., 2012). Interestingly, another TRP ion channel, TRPV4, changes in expression in a mouse model of OA (Lamandé et al., 2011) and knockout of TRPV4 ion channels in mice results in osteoarthritic changes in cartilage (Clark et al., 2010). This channel is therefore a potential biomarker for OA. Indeed, the closely related TRPV1 was also associated to OA pain in the genetic study mentioned above (Thakur et al., 2013). It remains to be seen whether modulation of TRP channel expression or function could be the basis of a plausible therapeutic approach for OA.

A further ion channel reportedly changed in activity in OA is the N-methyl-D-aspartic acid (NMDA)-receptor. This ion channel is better known as the most abundant excitatory neurotransmitter receptor in the brain. It is activated by glutamate and its presence in chondrocytes is indicative of the complex signaling between chondrocytes and the extracellular matrix (ECM). Several NMDA isoforms have been identified in chondrocytes; NR1, NR2A, NR2B, NR2D, and NR3 (Ramage et al., 2008; Lee et al., 2009; Piepoli et al., 2009) and stimulation of this channel has a number of biological effects, elevation of intracellular Ca^2+^, activation of nNOS, uncoupling of PDZ, depolarization and cell proliferation (Ramage et al., 2008; Piepoli et al., 2009). The depolarization can be reversed by application of the classical voltage-gated sodium channel blocker (tetrodotoxin). Following such treatment NMDA-receptor activation results in a partially apamin sensitive (i.e., SK mediated) hyperpolarization. The expression pattern of NMDA-R isoforms changes with onset of OA: RT-PCR studies show that chondrocytes from normal cartilage express mRNA for NR1 and NR2A. In the lysate samples tested by Ramage et al. OA chondrocytes showed decreased expression of NR2A, but increased expression of NR2B during onset of OA. Normal chondrocytes show very little proliferative potential, and it is interesting that IL1β activates gene expression in an NMDA-R manner. Therefore, NMDA-R may be involved in cartilage degradation in OA. In terms of soluble biomarkers, changes in membrane receptor expression could be predictive of changes in soluble partner ligand. It is therefore notable that changes in NMDA-R ligands (excitatory amino acids) are indeed altered in OA cartilage (dialysates) (Jean et al., 2005, 2006, 2007).

### Histamine receptors

Other membrane receptors are also differentially expressed in OA. These include histamine, bradykinin, and prostaglandin receptors, all linked to inflammation and extravasation in tissues. Histamine was probably the earliest pharmacological mediator to be discovered that is strongly linked to inflammation. Typically histamine is released at site of tissue injury and serves to initiate a local inflammatory response. Histamine antagonists are one of the most widely used off the shelf medicines; H_1_ antagonists have sedative value if they cross the blood brain barrier, but are useful anti-allergenics for hayfever etc., whereas H_2_ are inhibitors of gastric acid secretion and used in many widely available anti-ulcer medicines. The earliest discovery of histamine receptors in cartilage was indeed of H_1_ and H_2_ (Tetlow and Woolley, 2005). Expression of both, together with the histamine producing enzyme histidine decarboxylase was seen to be increased in expression, especially in the superficial zone of OA cartilage (Tetlow and Woolley, 2005). Physiologically, histamine increases intracellular calcium ion concentrations evoked by the ORAI/STIM1 pathway and so hyperpolarizes chondrocytes via BK channels (Funabashi et al., 2010; Inayama et al., 2015). Other studies of histamine receptors in cartilage have focussed on the less well-known H_4_ histamine receptor: Comparisons between rheumatoid and OA tissue show OA to have greater expression of this receptor (Yamaura et al., 2012b). This could be a key differential marker between the systemic inflammation seen in rheumatoid arthritis and the local inflammation of OA. A further explanation, which goes someway to explain this observation, is that histamine H_4_-R density is elevated in a teratoma-derived pre-chondrocyte cell line, ATDC5, (Yamaura et al., 2012a) providing more evidence of the apparent changes in chondrocyte phenotype as cartilage degenerates, even without inflammation.

### Prostaglandin receptors

In many ways, bradykinin and the prostaglandins are partners to histamine. They are both central to the early response to tissue injury and inflammation, and released in the tissues along with histamine. Prostaglandins are a family of lipid mediators derived from the cell membrane via the action of phospholipase A2 (PLA_2_) and cyclooxygenase (principally COX-1 and COX-2) enzymes. Inhibitors of these enzymes therefore profoundly change the prostaglandin balance in the joint. PLA_2_ is inhibited by steroid anti-inflammatory drugs and COX-1 and COX-2 are inhibited by typical NSAIDs. The prostaglandin membrane interactions documented to date surround prostaglandin E_2_ (PGE_2_) and its receptors EP_2_ and EP_4_ (Attur et al., 2008; Otsuka et al., 2009). This work has centered on a pharmacological, rather than molecular or immunohistochemical approach. PGE_2_ is an enzyme typically associated with pro-inflammatory actions (Martel-Pelletier et al., 2003), it binds preferentially to 4 receptors termed EP_1-4_ and there are a number of relatively selective agonists and antagonists available to distinguish between them (Alexander et al., 2011). PGE_2_ membrane receptors have been reported to have diverse actions on OA cartilage, for, whilst the EP_4_ receptor stimulates activity of a number of catabolic enzymes in OA cartilage (Attur et al., 2008), there are conflicting reports of the actions of EP_2_-receptor activation. Activation of PGE_2_ EP_2_ receptors was reported to decrease proteoglycan secretion in 3D cultures of human articular chondrocytes (Li et al., 2009), but be protective (Mitsui et al., 2011) and to even enhance regeneration of articular cartilage in rabbit OA models (Otsuka et al., 2009). The mechanism of action of each of the PGE_2_ chondrocyte membrane receptors has not been well studied, however, a role in intracellular Ca^2+^ handling has been proposed (Xu et al., 2009). Chondrocyte activity is controlled by a complex mechanotransduction mechanism, beginning with the deformation of the cellular membrane and ending with changes in biosynthetic activity. This pathway involves opening of ion channels, change of membrane potential and elevation of intracellular Ca^2+^. Xu et al. (2009) mimicked this biological activity with direct electrical stimulation of cartilage and found the ECM synthesis output was dependent on COX activity (i.e., indomethacin sensitive). Whilst it was inferred that PGE_2_ receptors were involved this was not tested for with a specific EP-receptor antagonist. Again such involvement of membrane receptors such as the EP_2_ and EP_4_ in pathogenesis of OA presents not just the receptor itself as a potential biomarker (and treatment target), but suggests changes in the associated ligand (PGE_2_) may also be detectable. Indeed this proves to be the case, since COX-2 and PGE_2_ are both found to be increased in cartilage from OA donors (Amin et al., 1997).

### Bradykinin (kinin) receptors

Bradykinin involvement with OA progression was first postulated by the group of Maggi (Meini and Maggi, 2008). Bradykinin contributes to the hyperexcitability of sensory nerves associated with inflammation and also activates the synoviocytes and chondrocytes critical for the homeostasis of synovial fluid and cartilage, respectively. Two bradykinin GPCRs have been identified; B_1_ and B_2_ and both signal through G_q∕11_. Typically bradykinin itself acts on B2 receptors. A polymorphism in the gene encoding this receptor is associated with OA, and could potentially be a biomarker for OA (Chen et al., 2012). Receptor antagonists toward B2 receptors have been proposed to be potential treatments for OA (Meini et al., 2011) since they can be protective to cartilage in *in vitro* models. A role of B2 membrane receptors in pathogensis implies a likely change in bradykinin levels too. This was observed many years ago, however, the change is seen in rheumatoid arthritis too and is not limited to OA (Melmon et al., 1967; Eisen, 1970). The contribution of B2-receptors to the pain and hyperalgesia of osteoarthritis is likely to be mediated by the same mechanisms of other chronic pain conditions, namely the sensitization of primary afferent neurons (Cesare and McNaughton, 1996; Huang et al., 2006), for example by increasing the neuronal expression of TRPV1 like channels via a protein kinase C (PKC) dependent pathway. The mechanisms by which B2-R facilitates cartilage breakdown are less well known, however, stimulation of prostaglandin synthesis and activation of EP2 receptors seems likely (Averbeck et al., 2004).

### Purinergic receptors

There are four members of the purinergic “P1” subfamily; A_1_ and A_3_ are G_i∕o_ coupled receptors, but A_2A_ and A_2B_ are coupled to G_s_. The P2 family are activated by extracellular ATP or ADP and further classified into 2 subfamilies P2X and P2Y. P2X are ligand-gated ion channel receptors (P2X_1−7_) with P2X_7_ being genetically linked to OA pain (Thakur et al., 2013). P2Y are G-protein coupled receptors. There are 14 known members of the P2Y class of membrane receptor and they couple to a variety of different G-proteins, including both G_s_ and G_i_. Several members of this superfamily have been identified in chondrocytes, including; P1 receptor A_2A_ and P2 receptors P2X_2_, P2X_3_ P2X_4_, P2X_7_, and P2Y_1_ and P2Y_2_ (Varani et al., 2008; Knight et al., 2009; Campo et al., 2012b). Most of these receptors are identified throughout cartilage with the notable exception of P2Y_2_ which was located to the superficial layers (Knight et al., 2009). Knight found there to be no obvious difference in transcription, but observes that since ATP/P2 receptor signaling is altered in OA elevated levels of ATP could be inducing desensitization of purinergic membrane receptors. In terms of function, there are clear indicators that the P2 receptor is involved with mechanotransduction coupling, in complex cascades including connexins and integrins (Millward-Sadler et al., 2004; Pingguan-Murphy et al., 2006; Knight et al., 2009), whereas an anti-inflammatory role has been proposed for the P1 (adenosine) receptors (Campo et al., 2012a). An important study by Rosenthal et al. (2010) indicates that P1 and P2 purinergic receptors interact to modulate the extracellular inorganic pyrophosphate balance and it seems likely that this too will be in response to mechanical activity, but this has not yet been shown.

### Estrogen receptors

There are two fundamentally different types of estrogen membrane receptor; one is a seven transmembrane (7TM) G-protein coupled “GPE” receptor. The GPE-receptor couples to both Gs and Gi/o and is thought to mediate rapid, so called non-genomic actions of estrogen. These may be localized in either plasma or intracellular membranes. The second are the steroid intracellular receptors known as ERα and ERβ. Both ERs have been identified in joint tissue including chondrocytes (Ushiyama et al., 1999; Martin-Millán and Castañeda, 2013). There have been conflicting reports of association between estrogen receptor gene polymorphisms and human OA. Association between ERα and ERβ was detected in a study of knee and hip OA (Riancho et al., 2010), whereas polymorphisms of ERβ were reported to be only marginally associated with OA in a study of hand and hip OA (Kerkhof et al., 2010). A large transgenic study showed that mice with double knock-out of both ERα and ERβ were strongly predisposed toward osteophyte formation, however, interestingly this was not the case if only ERα or ERβ were genetically deleted (Sniekers et al., 2009). Note that the estrogen-related receptor is also dysregulated in OA (Bonnelye et al., 2011). The decrease of estrogen levels decrease in older women could therefore contribute to OA, however estrogen replacement provides only modest, and variable improvements to patients (Roman-Blas et al., 2009). Exactly, how estrogen receptors affect joint pathology is not known, but they induce changes in many ECM proteins aggrecan-1, MMP1, MMP2, MMP13, MMP14, and TIMP2 (Claassen et al., 2010; Sniekers et al., 2010) together with some anti-inflammatory action (Martin-Millán and Castañeda, 2013). Both the selective estrogen receptor modulator raloxifene and 17β-estradiol reduce chondrocyte apoptosis induced by TNFα (Hattori et al., 2012) or doxorubicin. The mechanism of action here has been investigated in some detail with ERK1/2 signaling and inhibition of volume sensitive chloride channels both implicated. The molecular identity of the volume sensitive chloride channel has not yet been confirmed.

## Conclusion

**Figure 1:**
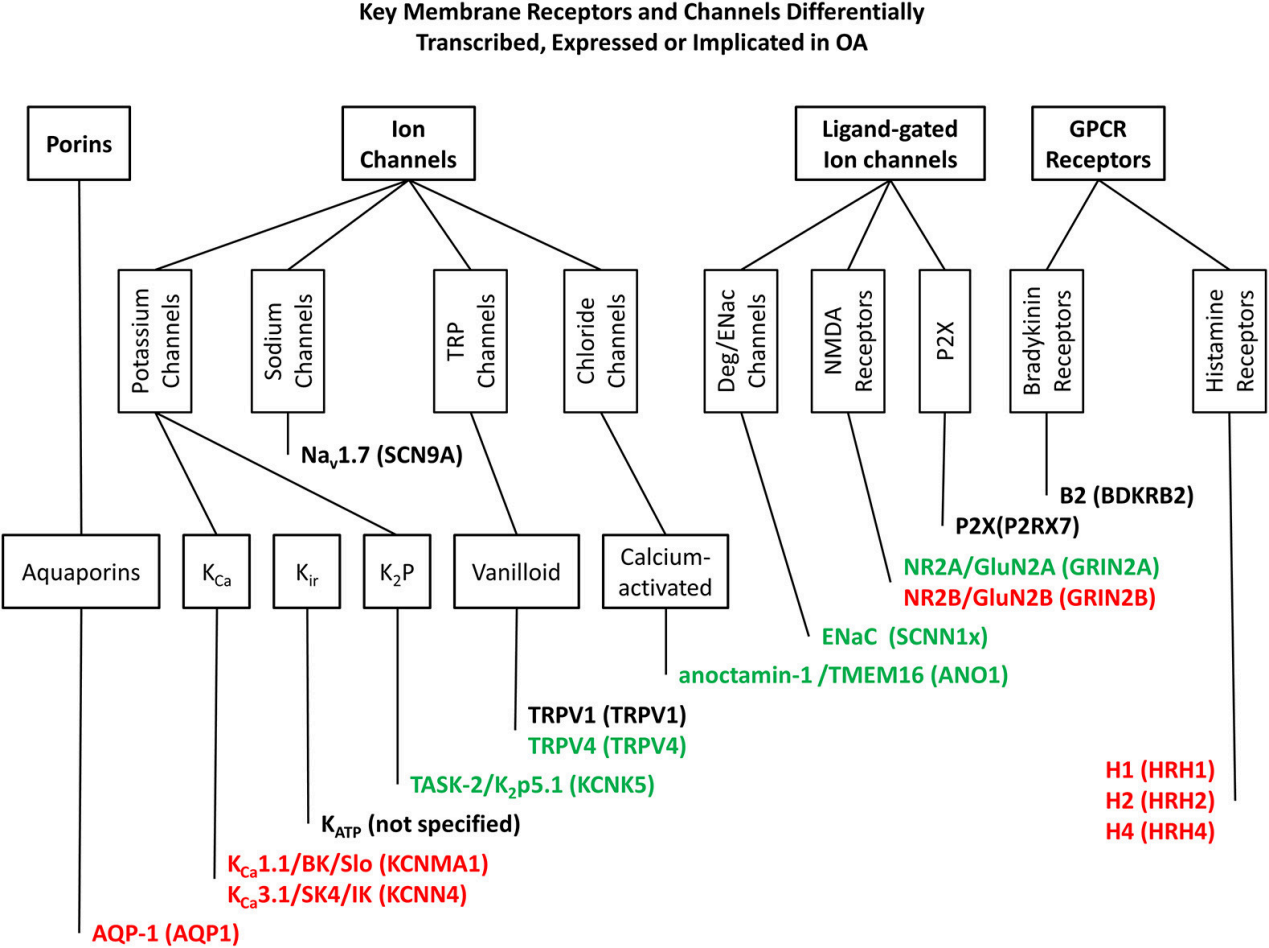
**Key membrane receptors and channels differentially transcribed**. Expressed or implicated in OA. This figure shows those ion channels and membrane receptors most clearly linked to OA and discussed in the text, classified in accordance with the IUPHAR Guide to Pharmacology database (Pawson et al., 2014). Genes/proteins in red are up regulated in OA, and those in green are down regulated. Those remaining in black are linked to OA, but not specifically increased or decreased (a polymorphism for example). Genes/proteins are given in the following format; common names/alternative names (human gene or equivalent).

Chondrocytes respond to mechanical signals by changing the production of cartilage components. However, the mechanisms of this process are understood only in outline. Membrane receptors and ion channels are well placed to transduce these signals and their differential expression, or post-translational dysfunction could contribute to the progression of OA and other degenerative joint conditions. This review has highlighted a number of such potential biomarkers, the most well established data are summarized in Figure 1 and further examples of membrane proteins, with less established but distinct associations with OA are given in Table 1. Many membrane receptors and ion channels altered in either expression and/or function in OA tissue and these may form the basis for biomarker discovery as well as providing deep insight into the mechanisms of cartilage production and provide the basis for development of future biologics for the treatment of degenerative joint conditions.

**Table 1:** **Other membrane receptors associated or implicated with osteoarthritis, but with less well characterized roles in joint function**.

**Receptor**	**Long name or descriptor**	**Context**
LRP-1	Low-density lipoprotein receptor-related protein (aka apolipoprotein E receptor, CD91), reviewed by May et al. (2007)	Drives rapid endocytosis of ADAMTS-5; LRP-1 is *down-regulated* in human articular cartilage from OA patients. Transcript abundance increased in damaged vs. intact cartilage (Geyer et al., 2009)
InsR/IGF-R	Insulin Receptors and Insulin like Growth factor receptors	Both InsR/ILGF-R are decreased in chondrocytes from patients with OA (Rosa et al., 2011) and the IGF-I peptide itself increases expression of COL2A1 in articular chondrocytes (Renard et al., 2012)
TLR	Toll-like receptor proteins (members of the interleukin receptor family), with many ligands such as heat shock proteins and hyaluronic acid oligomers. Includes TLR_1−11_ (Akira and Takeda, 2004)	Are more commonly found on inflammatory cells and serve to initiate inflammatory responses/innate immunity. TLR_1−9_ identified in cartilage and are differentially expressed in OA cartilage (Kuroki et al., 2010; Barreto et al., 2013; Yang et al., 2013). Polymorphism of the TLR_3_ and TLR_9_ promoted associate with severe OA (Su et al., 2012; Yang et al., 2013) and “alarmins” accelerate catabolism in OA cartilage in a TLR4 dependent mechanism (Schelbergen et al., 2012)
TGFβ-RII	Transforming growth factor β receptor 2	TGFβ-RII expression decreases with age and potentially predisposes older people to OA (Bauge et al., 2013) and its genetic deletion increases OA in mice (Zhen et al., 2013)
PPARγ-R	Peroxisome Proliferator-Activated Receptor-γ, a nuclear receptor	PPARγ-R is down-regulated in both human OA (Afif et al., 2007) and in several OA models (Fahmi et al., 2011) including the spontaneous Guinea-pig model (Nebbaki et al., 2013). In some cases this is secondary to Erg-1 mediated IL-1 (Nebbaki et al., 2012) and/or TLR_4_ activation (Chen et al., 2013)
MC-R	Melanocortin peptide receptors, for example MC_1_-R and MC_2_-R. Endogenous pharmacological activators include α-melanocyte-stimulating hormone (α-MSH), pro-opiomelanocortin (POMC) and adrenocorticotrophin (ACTH)	MC1, MC2, MC5, and ligand POMC transcribed in cartilage, induces expression of several ECM components and pro-inflammatory cytokines (Grässel et al., 2009), but can also mediate chondro- and cartilage- protective effects of neuropeptides such as α-MSH, POMC in both *in vitro* and rat *in vivo* models of OA (Shen et al., 2011; Kaneva et al., 2012)
CD36	A pattern recognizing receptor (Silverstein and Febbraio, 2009). Aka “thrombospondin receptor”	CD36 is well known to increase in OA (Pfander et al., 2000), however, recent data show it is also able to suppress catabolic activity and serves as a marker of hypertrophy (Cecil et al., 2009)
PTH1-R	Parathyroid receptor-1	PTH1-R expression is decreased in rabbits with ACL section induced OA (Becher et al., 2010)
Ob-R	Leptin receptor	With onset of OA there is a switch from adipokine synthesis to receptor synthesis (Francin et al., 2011). Leptin itself enhances production of catabolic MMP enzymes in OA cartilage (Iliopoulos et al., 2007; Koskinen et al., 2011). It is believed that leptin may mediate the pro-OA effects of obesity rather than simply the increased load observed in weight baring joints
CD44	Hyaluronan receptor (aka HA-R)	Activation inhibits expression of ADAMTS4 (aggrakinase-1) and MMP-13 in [osteoarthritic] chondrocytes (Yatabe et al., 2009; Julovi et al., 2011), although some hyaluronan effects could be mediated through the TLRs (*see below*)
FGF-R	Fibroblast growth factor (FGF) receptors, family includes FGFR1, 2, 3, and 4. Sensitive to the 22 member FGF family	FGFR-3 in has been shown to mediate chondroprotective of FGF18 (Ellman et al., 2013). Please see Vincent (2012) for discussion of the role of FGF in OA.

## Conflict of interest statement

The authors declare that the research was conducted in the absence of any commercial or financial relationships that could be construed as a potential conflict of interest.
